# Antimicrobial Peptides: Versatile Biological Properties

**DOI:** 10.1155/2013/675391

**Published:** 2013-06-26

**Authors:** Muthuirulan Pushpanathan, Paramasamy Gunasekaran, Jeyaprakash Rajendhran

**Affiliations:** ^1^Department of Genetics, Centre for Excellence in Genomic Sciences, School of Biological Sciences, Madurai Kamaraj University, Madurai 625 021, India; ^2^Thiruvalluvar University, Vellore 632106, India

## Abstract

Antimicrobial peptides are diverse group of biologically active molecules with multidimensional properties. In recent past, a wide variety of AMPs with diverse structures have been reported from different sources such as plants, animals, mammals, and microorganisms. The presence of unusual amino acids and structural motifs in AMPs confers unique structural properties to the peptide that attribute for their specific mode of action. The ability of these active AMPs to act as multifunctional effector molecules such as signalling molecule, immune modulators, mitogen, antitumor, and contraceptive agent makes it an interesting candidate to study every aspect of their structural and biological properties for prophylactic and therapeutic applications. In addition, easy cloning and recombinant expression of AMPs in heterologous plant host systems provided a pipeline for production of disease resistant transgenic plants. Besides these properties, AMPs were also used as drug delivery vectors to deliver cell impermeable drugs to cell interior. The present review focuses on the diversity and broad spectrum antimicrobial activity of AMPs along with its multidimensional properties that could be exploited for the application of these bioactive peptides as a potential and promising drug candidate in pharmaceutical industries.

## 1. Introduction

The antimicrobial peptides (AMPs) are biologically active molecules produced by wide variety of organisms as an essential component of their innate immune response. The primary role of the AMPs is host defense by exerting cytotoxicity on the invading pathogenic microorganisms, and they also serve as immune modulators in higher organisms [1]. AMPs are considered as a promising and potential drug candidate for the future due to their broad range of activity, lesser toxicity, and decreased resistance development by the target cells [2]. The AMPs were found to exist in a wide range of secondary structures such as *α*-helices, *β*-strands with one or more disulphide bridges, loop and extended structures. The existences of such diverse structural forms of AMPs are highly essential for their broad spectrum antimicrobial activity [3]. Besides these properties, certain crucial factors such as size, charge, hydrophobicity, amphipathic stereo geometry, and peptide self-association to the biological membrane also attributes for their broad spectrum antimicrobial activity. The smaller size of AMPs facilitates the rapid diffusion and secretion of peptide outside the cells, which is required for eliciting immediate defence response against pathogenic microbes [4]. The differences in the lipid composition between prokaryotic and eukaryotic cell membranes represent the targets for AMPs. The antimicrobial specificity of AMPs towards the target cells was highly dependent on the preferential interaction of peptides with the microbial cells, which enable them to kill specific target cells without affecting the host cells [5]. In addition, net charge and hydrophobicity of AMPs play a crucial role in cellular association of these peptides to selective target cellular membranes in exerting antimicrobial activity [6]. AMPs have been reported from different sources such as plants, mammals, insects, marine invertebrates, and environmental libraries (Table 1). Currently, more than 2,000 AMPs have been reported in antimicrobial peptide database (http://aps.unmc.edu/AP/main.php/). Most of them are cationic peptides, and only a few of them are anionic, which shared the ability to fold into amphipathic conformation upon interacting with the membranes [7]. Besides antimicrobial function, AMPs also serve as drug delivery vectors, antitumor agents, mitogenic agents, contraceptive agents, and signalling molecules in signal transduction pathways [8]. This review provide insight into antimicrobials as well as multifunctional properties of AMPs that provides better understanding of versatile biological properties of AMPs for prophylactic and therapeutic application.

## 2. Antimicrobial Properties of AMPs

### 2.1. Structural Features of AMPS

AMPs are generally defined as peptides of less than 100 amino acid residues with overall net charge of +2 to +9, represented by positively charged amino acids such as lysine and arginine along with a substantial portion of hydrophobic residues. The structural and physicochemical properties of AMPs play an essential role in determining its specificity towards the target cells. The antimicrobial peptides with different structural forms were listed in Table 2.

#### 2.1.1. Structural Motifs of AMPs

Antimicrobial peptides have served a fundamental role in evolution of complex multicellular organisms [31]. Insight into the conserved structural elements of AMPs provides information regarding the evolutionary significance of AMPs that serves as template for the design of novel peptide antibiotics [32]. The antimicrobial peptide with proline-arginine-proline (PRP) motif includes proline/arginine-rich cationic peptides, callinectin, and astacidin 2. These peptides contain one or more PRP motif, which showed potent antibacterial activity against Gram-positive and Gram-negative bacteria. Armadillidin is a glycine rich, cationic antimicrobial peptide with unique five-fold repeated motifs of GGGFHR or GGGFHS and amidation at the C-terminal end that displayed potent antibacterial activity against Gram-positive bacteria [33]. Penaeidins are chimeric cationic peptides that consist of PRP motifs at the N-terminal end (PRP-domain) and cysteine rich region at the C-terminal end (cysteine-rich domain) with a conserved chitin binding motif that possessed antimicrobial activity against Gram-positive bacteria and fungi [34]. Crustins are cationic antimicrobial peptides with specific antibacterial activity against Gram-positive bacteria. It possessed a Wap domain at the C-terminal region with eight conserved cysteine residues forming four disulphide core (4DSC), which was highly responsible for protease inhibitory or regulatory mechanisms [35]. Kalata, circulin A & B and cyclopsychotride are macrocyclic cysteine knot-peptides with end-to-end macrocycle and cysteine-knot motif that displayed potent antimicrobial activity against Gram-positive, Gram-negative bacteria, and yeast [36]. Disulphide bridge containing antimicrobial peptides such as HNP-3, mBD-8, phormicin, drosomycin, Ah-AMP-1, MGD-1, protegrin-1, big defensin, gaegurin-1, tachyplesin-1, polyphemusin-1, mytilin A, gomesin, thanatin, and AFP-1 contain conserved GXC or CXG motif (*γ*-core signature) [32]. The AMPs with heparin binding ability contained heparin binding motif such as XBBBXXBX or XBBXBX (where X represents hydrophobic or uncharged amino acids, and B represents basic amino acids). The LL-37 is an amphipathic peptide with helical structure and XBBXBX motifs responsible for heparin binding property [37]. Certain AMPs such as penaeidin, tachystatin, Cy-AMP, Ee-CBP, and MMGP1 contain conserved cysteine residues at the C-terminal ends responsible for chitin binding activity [38].

#### 2.1.2. Structural Requirements and Modification of AMPs

The structural and physicochemical properties of AMPs play an important role in conferring specific toxicity against the target cells. Tachystatin is an antimicrobial peptide identified from the hemocytes of the horseshoe crab, *Tachypleus tridentatus*, which showed broad spectrum of antimicrobial activity against Gram-positive, Gram-negative bacteria, and fungi. The amphiphilic *β*-sheet at the C-terminal end of tachystatin was highly responsible for their cytolytic activity against the target cells [39]. Tenecin 1 is an inducible antibacterial peptide from larvae of *Tenebrio molitor,* with *α*-helical and *β*-sheet fragments at their C-terminal and N-terminal ends that displayed potent antimicrobial activity specifically towards Gram-positive bacteria. The chemical synthesis of truncated peptides revealed that the C-terminal *β*-sheet domain of tenecin 1 is highly responsible for both the antifungal and antibacterial activity. The fragment corresponding to *α*-helical region did not show any antimicrobial activity. This might be due to difference in the net positive charge of both *α*-helical (+1) and *β*-sheet region (+5) [40]. Thanatin is an inducible antimicrobial peptide reported from the insect *Podisus maculiventris *with a broad spectrum of antimicrobial activity against Gram-positive, Gram-negative bacteria, and fungi. The mechanism of action of thanatin involved binding of peptide to the target cell membrane and thereby reduced the surface charge density of lipopolysaccharide and the electrostatic repulsion between the cells, which cause rapid aggregation of target cells and cell death [41]. Unlike antibacterial peptides, no conserved structural domains have been reported for antifungal peptides. Most of the antifungal peptides have been reported with chitin and heparin binding abilities [41]. The antimicrobial peptides entered the cells through energy dependent or energy independent mechanisms. The AMPs rich in positively charged amino acids such as arginine and lysine induced energy dependent endocytic pathway such as macropinocytosis for entering into the cells, whereas other AMPs such as MMGP1 and maganin entered the cells through energy independent direct cell-penetration mechanism, which does not require ATP [42–44]. 

Most of the naturally occurring AMPs are not optimised for efficient activity and need to be improved through different strategies, before it could be used as therapeutics. Recently, several methods have been tested using the native templates to generate more efficient AMPs such as random mutagenesis, quantitative structure-activity relationship (QSAR), altering the peptide structures by cyclization, or by increasing the charge or hydrophobicity of the peptide by tagging. Random mutagenesis involved methods that modify the naturally existing AMPs by addition/deletion/replacement of single or more residues or truncation at the N- or C-terminal or generation of chimeric peptides by combination of both methods [56]. QSAR provides a working conceptual model of bioactive peptides, which attempt to find consistent relationship between biological activity and molecular properties. AMPs based QSAR studies involve limited set of systemic modification of residues in naturally occurring AMPs to form peptides with amphipathic structures. In this, a few aminoacids with specific characteristics such as basic (lysine or arginine) or hydrophobic amino acids (alanine, leucine, phenylalanine or tryptophan) are used to obtain peptide with maximum activity and minimum toxicity towards the host [57]. Further, the chemical attachment of aliphatic acids to the N-terminus of biologically inactive peptides with different lengths (10, 12, 14, and 16 aa) resulted in generation of lipopeptides with lytic activity. The selectivity of these lipopeptides against bacteria, fungi, and human erythrocytes was influenced by the length of fatty acids chain, which attributed for increased antimicrobial activity and resistance to proteases [58]. In addition, chemically induced cross links or introduction of covalent lactam bonds in AMPs provided a way for introducing conformational constraint in peptides that confer newer properties to AMPs. The formation of covalent cross link between Trp 6 and Trp 9 in synthetic indolicidin analogue showed decreased susceptibility to protease. Similarly, the formation of covalent lactam bond between cecropin-melittin hybrid peptide improved its antibacterial activity [59]. The antimicrobial activity of AMPs could also be enhanced through modification of their existing structural forms by manipulating the hydrophobicity or flexibility of the peptide secondary structures. The antimicrobial activity of antibacterial peptide, indolicidin isolated from bovine neutrophils was increased by bringing the C-termini and N-termini regions of peptide closer to each other, and the modified peptide was stabilized with disulphide bond by introducing cysteine residues at both the ends, which showed enhanced antibacterial activity against Gram-negative bacteria [60].

#### 2.1.3. AMPs with Unusual Amino Acids

**Figure 1:**
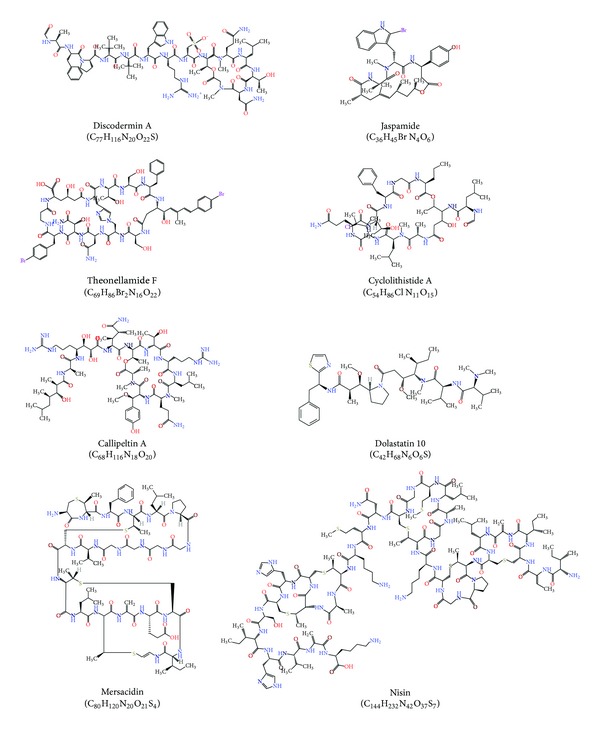
Representative chemical structure of AMPs with unusual amino acids.

Many AMPs have unusual amino acids and hence have unusual structures, which attributed for wide range of bioactivities (Figure 1 and Table 3). Depsipeptides are nonribosomal peptides characterized by one or more of the amide (–CONHR–) bonds replaced by an ester bonds (COOR). Depsipeptides also contained organic acids in addition to amino acids. Examples for depsipeptides include discodermin A, jaspamide, theonellamide F, cyclolithistide A, callipeltin A, dolastatin 10, and theonegramide [61]. Lantibiotics are ribosomal synthesized antibacterial peptides produced by some Gram-positive bacteria and are characterized by the presence of unusual amino acids such as lanthionine and dehydrated amino acid dehydroalanine and 2-aminoisobutyric acid, which includes nukacin ISK-1, mersacidin, microbisporicin, lacticin 3147, planosporicin, and nisin [62].

### 2.2. Mechanisms of Action of AMPs

AMPs have attained dynamic interchange in their structure and topologies upon interacting with the microbial cell membranes [63]. The outer surface of prokaryotic cell is negatively charged due to the presence of lipopolysaccharides or teichoic acid, whereas the outer leaflet of eukaryotic cell is composed of zwitterionic phosphatidylcholine and sphingomyelin phospholipids [64]. The electrostatic interaction of peptides with the negatively charged molecules on the membrane seems to be the primary mechanism for antimicrobial activity. In other cases, AMPs exert antimicrobial activity in target cells by translocating across the cell membrane and inhibit essential cellular processes such as protein synthesis, nucleic acid synthesis, enzymatic activities, and cell wall synthesis [7]. Certain other factors such as magnitude and charge of the outer membrane, the concentration of negatively charged molecules, molecular architecture, and fluidity of the outer membrane were also essential for the transport of peptide across the membrane [65]. The fluidity of the membrane was found to regulate the adsorption and insertion of AMPs into the biological membrane. Based on the mechanisms of action, antimicrobial peptides are broadly categorized into membrane acting and nonmembrane acting peptides. Membrane permeabilizing peptides are mostly represented by cationic peptides capable of forming transient pores on the membrane, whereas nonmembrane permeabilizing peptides have the ability to translocate across the cell membrane without permeabilizing the membrane. Certain antibacterial peptides forming transmembrane pores on the target cell membrane include defensin [66], melittin [67], magainins [68], and LL-37 [69]. Antimicrobial peptides such as buforin II [44], dermaseptin [70], HNP-1 [71], pleurocidin [70], indolicidin [26], pyrrhocidin [23], and mersacidin [52] get translocated across the cell membrane and inhibit essential cellular processes that lead to cell death. Certain antifungal peptides such as papiliocin [72], melittin [73] histatin [74], and lactoferrin [70] exert their antimicrobial action through formation of reactive oxygen species. 

**Figure 2:**
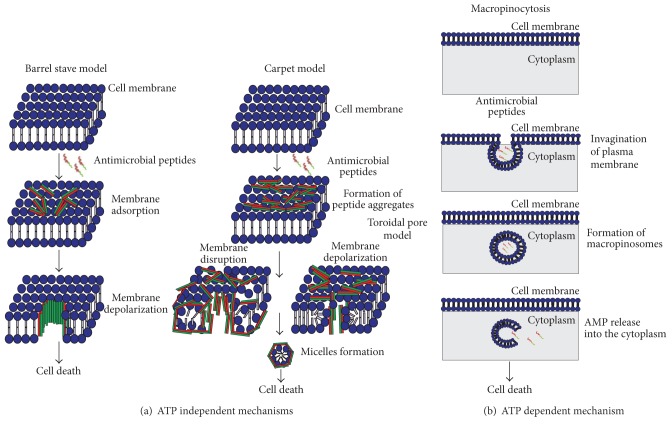
Proposed mechanisms of actions of AMPs. (a) Energy independent mechanism: it includes barrel stave model, carpet model, and toroidal pore model. (b) Energy dependent mechanism: it includes macropinocytosis.

AMPs promote membrane damage in target cells either by membrane thinning or by pores formation or by lipid bilayer disruption [75]. Several models have been proposed to describe the mechanism of action of antimicrobial peptides. The cellular uptake mechanisms of AMPs are categorized into energy dependent and energy independent uptake mechanisms (Figure 2). Energy independent uptake mechanisms include barrel-stave model, carpet model, or toroidal model, and energy dependent uptake mechanism includes macropinocytosis. In barrel-stave mechanism, the peptide monomers get aggregated on the surface of the membrane. The aggregated peptides get inserted into the membrane and orient themselves in such a way that their nonpolar side chains direct the hydrophobic lipid core of the membrane, and the hydrophilic surfaces of peptides point inward and formed water filled transmembrane pore that caused release of intracellular content and consequent cell death. An example for antimicrobial peptides that follows barrel-stave mechanisms includes alamethicin and gramicidin S [76–78]. In carpet model, the peptides initially get associated on the surface of the membrane and form a local carpet. Once particular threshold concentration was reached, the peptide induced membrane permeation that leads to disruption of cell membrane and causes lysis of the microbial cells [79]. In toroidal pore model, the aggregated peptides either prior or after binding with the membrane surfaces induced membrane depolarization and form a toroidal shaped transmembrane pores with micellar formation that leads to cell death [80]. Macropinocytosis is the energy independent uptake route of AMPs, in which the plasma membrane of the target cells folds inward along with the peptide and forms vesicles called macropinosomes. Subsequently, the AMPs within the vesicles get released into the cytoplasm and exert their antimicrobial action [81].

## 3. Multidimensional Properties of AMPs

### 3.1. AMPs As Drug Delivery Vector

Nonlytic cell-penetrating AMPs were used as drug delivery vector to treat and manage several diseases. Certain large hydrophilic drugs cannot easily penetrate through the cell membrane barriers. In such cases, AMPs with efficient membrane translocating property, which could enter the cells without causing damage to the membranes, were used as drug delivery vectors [82]. The main feature of AMPs to serve as delivery vector is that they should be able to penetrate the cell membrane at very low concentrations (micromolar) without any specific receptors and capable of efficiently delivering electrostatically or covalently bound biologically active cargoes such as drugs into the cell interior [83]. Antibacterial peptides such as LL-37, TP10, and pVEC were associated with bacterial membrane damage shown to act as cell penetrating peptides (CPPs) without exhibiting toxicity to eukaryotic host cells [84, 85]. Representative analogue of antimicrobial peptides magainin 2 and buforin 2 was found to enter the human carcinoma cells through membrane translocating mechanisms. The translocation of magainin 2 analogue required transient pore formation as an intermediate steps, which showed higher toxicity to the carcinoma cells, whereas buforin 2 analogue translocated across the membrane in a less concentration dependent passive mechanism without causing significant toxicity to carcinoma cells [86]. SynB vectors are new family of peptide vectors derived from an antimicrobial peptide protegrin-1 (PG-1), lacking the cysteine residues responsible for membrane disrupting activity in protegrin-1. SynB vectors are capable of transporting very large molecules such as streptavidin (MW: 60 kDa) and IgGs (MW: 150 kDa) and were used to deliver drugs efficiently into complex biological membranes such as blood-brain barrier [87]. Pyrrhocoricin and Bac7 are cell-penetrating antimicrobial peptides that translocate across the cell membrane through binding of receptors [88, 89]. Certain antimicrobial peptides such as tat, penetratin, pep1, and MMGP1 were reported to enter the target cells through energy independent direct cell-penetration mechanisms [43, 90].

### 3.2. Tumoricidal and Mitogenic Properties of AMPs

**Figure 3:**
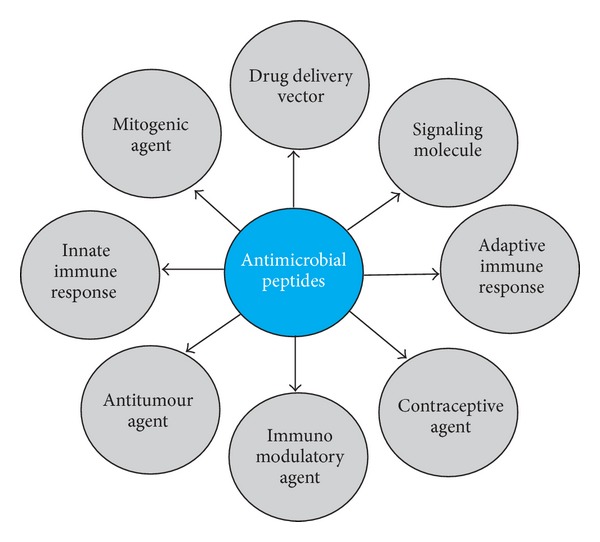
Schematic representation of multifunctional properties of antimicrobial peptides.

The ability of AMPs to interact with different cell membranes makes it to serve as the multifunctional effector molecules (Figure 3). Increased susceptibility of tumour cells to cationic membrane active AMPs due to the presence of high content of anionic phosphatidylserine molecules on their membranes than the normal cells makes it an interesting candidate to use AMPs as antitumour agents [110]. The selected AMPs with tumoricidal properties are listed in Table 4. AMPs such as magainins [111], defensins [112], BMAP-27 and BMAP-28 [113], gaegurins [114], tachyplesin I [115], cecropins, and melittin [99] were reported to exhibit tumoricidal activity against melanoma and carcinoma cells both under *in vitro* and *in vivo* conditions. Generally higher concentration of AMPs is required to achieve tumoricidal activity. For instance, magainin II (MG2) exhibited cytotoxicity in tumour cells only at higher concentration, likely due to the inefficiency of MG2 in cell membrane binding and its subsequent entry. Conjugation of CPP penetratin (Antp) to MG2 showed enhanced cytotoxicity to tumour cells at a lesser concentration [116]. Furthermore, AMPs are more susceptible to degradation by proteases in the extracellular matrix of the tumour cells, which leads to loss in their tumoricidal activity. This could be overcome by expression of AMP encoding gene directly into the tumour cells or by replacement of peptide amino acids by their D-amino acids and modification of peptide terminal by amidation [117]. Recent synthesis of truncated fragments of antibacterial peptides such as epinecidin-8 and pardaxin-6 showed higher tumoricidal activity against human epithelial carcinoma (HeLa) and fibrosarcoma (HT-1080) cell lines [118]. The combination of cell-penetrating-*γ* peptide, PEG-1, with antimicrobial undecapeptides showed efficient anticancer properties against MDA-MB-231 human breast cancer cells [119]. Certain AMPs such as pexiganan MSI-78, citropin 1.1, protegrin 1, synthetic lipopeptide, and N-*α*-palmitoyl-L-lysine–L-lysine amide (Pal-Lys-Lys-NH_2_) showed cytotoxic activity against U937 histiocytic cell line. Of these, pexiganan MSI-78, protegrin 1, and lipopeptide showed increased tumoricidal activity due to their stronger membranolytic activity that leads to necrosis [120]. Cecropins A and B showed selective inhibitory and antiproliferative efficacy against bladder tumour cells lines, RT4, 647V, J82, 486P, and benign fibroblast cell line, 3T6 [101]. Defensin stimulated the growth of normal fibroblast and epithelial cell under *in vitro* conditions, which was highly essential for the healing wounds under *in vivo* conditions. Dermaseptin (Drs) B2 is an AMP identified from the skin secretion of the Amazonian tree frog; *Phyllomedusa bicolor* had both antitumour and angiostatic activities against prostate adenocarcinoma cell line, PC3 in a xenograft model *in vivo* [92]. These functional dualism of AMPs to act as antitumor and mitogenic agent makes it an interesting candidate to study every aspect is of their biological activity for clinical applications. 

### 3.3. AMPs As Signalling Molecules

Host defense peptides (HDPs) are short cationic AMPs produced by the immune systems of most organisms, which plays a crucial role in innate immunity [127]. Most HDPs are involved in modulation of immune response as host defense and also act as modulators of signal transduction pathways by influencing the activity of intracellular signalling targets such as protein kinases (Table 5). Defensins are HDPs produced by different cell types such as lymphocytes, neutrophils, tissue macrophages, small intestinal epithelial cells, keratinocytes, and cardiomyocytes and are classified into two groups such as *α*-defensins and *β*-defensins. Defensins were known to involve in host cell receptor interaction, chemo attractant of immune cells, recruitment of neutrophils, mobilization of immunocompetent T-cells as well as enhancer of cell adhesion, and activation of classical complement pathways [129–133]. Especially, murine defensins regulate the migration and recruitment of antigen presenting and immunocompetent cells by binding with CC-chemokine receptors during inflammatory and immunological responses. Guinea pig defensins induced adhesion of neutrophils and inhibit generation of superoxide anion during phagocytosis of complement-opsonized particle [134]. LL-37 is a host defense AMP produced by different cell types such as neutrophils, mast cells, monocytes, and macrophages that serve as a chemoattractant of neutrophils and mast cells, inhibit neutrophil and keratinocytes apoptosis, promote chemokine induction, angiogenesis, and stimulate differentiation of monocytes and proliferation of vascular endothelium. In addition, it also exhibits anti-inflammatory and antiendotoxic effects [127]. PR-39 is a proline and arginine rich antimicrobial peptide isolated from pig intestine, which regulate various processes such as cell development, cell proliferation, cell cycle control, cell survival, migration, and invasion by binding with the Cas family adapter protein, p130 [135]. Besides antimicrobial and immune regulating action, AMPs play a key role in immune neuroendocrine interactions, taking part in the pathogenesis of stress reactions (corticostatic action) and also serve as regulatory peptides of adaptogenic action [136]. The epidermoid carcinoma—derived antimicrobial peptide (ECAP)—inhibits autophosphorylation of epidermal growth factor receptor and leads to decreased activity of Lyn and Syk tyrosine kinases [126]. AMPs at their subinhibitory concentrations activate numerous genes involved in signal transduction pathways. Sigma factors are an essential component of RNA polymerases and determine the selectivity of promoter. The substitution of one sigma factor for another can redirect RNA polymerases in a cell to activate the transcription of genes. The extra cytoplasmic function (ECF) sigma factors are small regulatory proteins that are quite divergent in sequence relative to most other sigma factors, and they function as antisigma factors that bind and inhibit cognate sigma factor upon receiving a stimulus from the environment [137] Naturally—derived AMP such as LL-37 and PG-1 serves as an activator of ECF sigma factors regulons such as SigW and SigM in a weak manner, whereas their synthetic analogue poly-L-lysine seems to be the strong activator of SigW [128]. SigM is required for maintaining the integrity of the cell envelope during stress induced by antibiotics, ethanol, heat, acids, and superoxides. It is also essential for the cells to survive under high salt concentrations [138, 139]. SigW is activated on stress induced by alkaline shock, inhibition of cell wall synthesis, disruption of membrane integrity by detergents [140, 141]. Certain immunomodulatory anti-infectives with antimicrobial properties in commercial development are CD-NP, a chimeric synthetic peptide NP (37 mer) used for the treatment of heart failure [142] and opebacan, Xoma 629 (Xoma), and CP-226 (Migenix), which could be used for the treatment of allogeneic stem cells transplantation-associated infections, endotoxemia in haematopoietic stem cells, impetigo, and catheter/dermatology-related infections [127].

### 3.4. AMPs As Contraceptive Agents for Vaginal Prophylaxis

A number of AMPs have been described in the reproductive tract of mammals that serves dual role on regulating fertility and preventing sexually transmitted diseases [143, 144]. Lactoferrin was found to be localized in the vaginal fluid and mucosal plug, which inhibit viral fusion and its subsequent entry by binding and disruption of the microbial membranes under acidic conditions. Cathelicidin was found to be present in mucosal secretions, vaginal secretions, and seminal plasma, which prevented the microbial infections following sexual intercourse by neutralizing the lipopolysaccharides of microbial cells. Defensins were reported to be present in ectocervix, vagina, testis, epididymis, seminal plasma, sperm, and germ cells that impair with the metabolic processes of microbes by penetrating the microbial membranes [145, 146]. Dermaseptins and magainins are two classes of cationic, amphipathic *α*-helical peptides identified in the skin extracts of frogs *Phyllomedusa sauvagei* and *Xenopus laevis*, which showed contraceptive activities against various sexually transmitted infections (STIs) causing pathogens and HIV infections [147]. Nisin possessed contraceptive effect by arresting the movement of spermatozoa, whereas magainin Al inhibited the sperm motility without causing damage to vaginal epithelial cells, thereby could be used as novel contraceptive microbicides [148, 149].

### 3.5. AMPs in Plant Transgenesis

Plants are constantly threatened by pathogenic microorganisms present in the environment. In recent, years, transgenic expression of genes encoding AMPs could help to enhance resistance against a wide range of phytopathogens. AMPs have been reported to be expressed in plant systems such as tobacco, banana, and potato for the production of pharmaceutical peptides and to develop transgenic plants that confer resistance to several plant diseases [17, 150]. The AMPs such as D4E1 [151], esculestin [73], MSI-999 [152], human lactoferrin [153], shiva-1, and SB-37 [154] were successfully expressed in plant systems that developed resistance against plant pathogens. A synthetic substitution analogue of antimicrobial peptide maiganin, MSI-99 imparts enhanced resistance to pathogenic fungi, *Aspergillus niger* in transgenic potato cultivars [155]. Integration of antimicrobial peptide genes *Np3* and *Np5* from Chinese shrimp (*Fenneropenaeus Chinensis*) into the rice plant, *Oryza sativa* L. subsp. *japonica cv. *Aichi asahi possessed broad spectrum resistance to rice bacterial blight disease [156]. Expression of a novel antimicrobial peptide penaeidin 4-1 from the shrimp, *Litopenaeus setiferus* in creeping bent grass, *Agrostis stolonifera *L. showed enhanced resistance to fungal disease, dollar spot, and brown patch [157]. Thus, the application of AMPs in plant transgenesis seems to be the alternative strategy for plant disease control. 

## 4. Conclusion

AMPs are potent agents with diverse structural and antimicrobial properties, which represent one of the most promising future drug candidate for combating infections and microbial drug resistance. In addition to their microbicidal activity, AMPs also possess other biological activities and have potential applications as signalling molecules, immune modulators, antitumour agents, drug delivery vehicles, and plant transgenesis mediators. Thus, understanding the versatile biological properties of AMPs can be of extreme importance for clinical development of peptide-based therapeutics.

## Figures and Tables

**Table 1:** Various sources of AMPs.

Source of AMPs	AMPs	References
Insect	Cecropin A, Sarotoxin IA, ponericin G2, ceratotoxin, stomoxyn, spinigerenin, thanatin, heliomicin, Alo3, sapecin, defensin A, smD1, gallerimycin, termicin, royalisin, drosomycin, drosocin, metchnikowin, apidaecin IA, abaecin, formaecin, lebocin, pyrrhocoricin, melittin, attacins, coleoptericin,diptericin,	[9, 10]
Amphibians	Japonicin-1 & 2, nigrocin 1 & 2, brevinin-20a, temporin-1Od, tigerin-1, pseudin-2, maximin-1, distinctin	[11]
Echinoderms	Strongylocins, centrocins, betathymosins, filamin A	[12]
Crustaceans	Callinectin, astacidin 2, armadillidin, homarin, scygonadin, penaeidin, crustin, hyastatin, arasin, stylicin, hemocyanin derived peptides	[13]
Plants	Thionins, plant defensins, lipid transfer proteins	[14]
Mammals	Defensin, histatin, LL-37, indolicidin, protegrin, lactoferricin	[15]
Bacteria	Iturin, bacillomycin, syringomycin, syringostatins, syringotoxins, nikkomycins	[16]
Fungi	Echinocandins, aculeacins, mulundocandins, FK463, aureobasidin, leucinostatins, helioferins	[17]
Fishes	Pardaxins, misgurin, pleurocidins, parasin, oncorhyncin II and III, chrysophsin and HFIAP	[18]

**Table 2:** List of antimicrobial peptides based on their structural features.

Class of AMP	Structural features	Representative peptides	Structure	References
Cationic peptides	Peptides forming *α*-helical structures	Cecropins	*α*-Helix	[19]
	Single disulphide bridge	Thanatin	*β*-Sheet	[20]
	Two disulphide bridge	Tachyplesin II	*β*-Sheet	[21]
	Three disulphide bridge	Penaeidins	*β*-Sheet	[22]
	More than three disulphide bridge	Drosomycin	*αβ*-Structure	[20]
	Proline-rich peptide	Pyrrhocoricin	*αβ*-Structure	[23]
	Glycine-rich peptide	Diptericins	—	[24]
	Histidine-rich peptide	Histatin	Rich in H	[25]
	Tryptophan-rich peptide	Indolicidin	Extended	[26]

Noncationic peptides	Neuropeptide derived molecules	Secretolytin	*α*-Helix	[27]
	Aspartic acid rich peptides	Dermcidin	—	[28]
	Aromatic dipeptides	*N*-Alanyl-5-s-glutathionyl- 3,4 Dihydroxy-phenylalanine and *p*-hydroxy cinnamaldehyde	—	[29]
	Oxygen binding proteins	Lactoferricin	*β*-Turn	[30]

**Table 3:** List of antimicrobial peptides with unusual amino acids.

Peptide	Source	Activity	Organisms	Unusual amino acids	References
Discodermin A	*Discodermia kiiensis *(sponge)	Antifungal	*C. albicans *	*Tert*-leucine (t-Leu), cystenoic acid, and sarcosine	[45]
Jaspamide	*Jaspis* sp. (sponge)	Antifungal	*C. albicans *	*N*-Methyl-2-bromo-D-tryptophan (Me-BrTrp) and L-*β*-tyrosine (L-*β*Tyr)	[46]
Theonellamide F	*Theonella *sp (sponge)	Antifungal	*C. albicans *	histidinoalanine, 3-Methyl-*p*-bromophenylalanine, (2*S*,4*R*)-2-amino-4-hydroxyadipic acid (L-Ahad), and (3*S*,4*S*,5*E*,7*E*)-3-amino-4-hydroxy-6-methyl-8-(*p*-bromophenyl)-5,7-octadienoic acid	[45]
Cyclolithistide A	*T. swinhoei *(sponge)	Antifungal	*C. albicans *	4-Chloroisoleucine (C1-Ile), 2-amino-pentanoic acid (D-Ape) and 4-amino-3,5-dihydroxyhexanoic acid (Adha)	[47]
Microsclerodermin A	*Theonella* sp. (sponge)	Antifungal	*C. albicans *	(2*S*,3*R*,4*S*,5*S*,6*S*,11*E*)-3-amino-6-methyl-12-(*p*-Methoxyphenyl)-2,4,5-trihydroxydodec-11-enoic acid (AMMTD), (3*R*)-4-amino-3-hydroxylbutyric acid (GABOB), and 3-hydroxy-4-amino-5-vinylpyrrolidone.	[48]
Callipeltin A	*Callipelta * sp. (sponge)	Antifungal Antiviral	*C. albicans*, HIV	(2*R*,3*R*,4*S*)-4-Amino-7-guanidino-2,3-dihydroxyheptanoic acid (AGDHE)	[49]
Dolastatin 10	*Dolabella auricularia *(sea hare), *Gymnangium regae *(Marine hydroid)	Antifungal	*Cryptococcus neoformans *	*O*-demethyldolaproline (Ddap), *N*-desmethyldolaisoleucine (Ddil), L-*threo*-phenylserine (L-Pser), and L-guanidino serine (Gser).	[50]
Nukacin ISK-1	*Staphylococcus warneri* ISK-1	Antibacterial	Gram-positive bacteria	Lanthionine, 3-methyllanthionine, and dehydrobutyrine	[51]
Mersacidin	*Bacillus subtilis *	Antibacterial	Gram-positive bacteria	Lanthionine or 3-methyllanthionine	[52]
Microbisporicin	*Microbispora* sp	Antibacterial	Gram-positive bacteria	5-Chloro-trypthopan and mono- (in A2) or bis-hydroxylated (in A1) proline	[53]
lacticin 3147	*Lactococcus lactis *	Antibacterial	Gram-positive bacteria	Lanthionine or *β*-methyllanthionine	[54]
Planosporicin	*Planomonospora *sp	Antibacterial	Gram-positive bacteria	Lanthionine and methyllanthionine	[53]
Nisin	*Lactococcus lactis *	Antibacterial	Gram-positive bacteria	Lanthionine methyllanthionine didehydroalanine didehydroaminobutyric acid	[55]

**Table 4:** List of antimicrobial peptides with antitumour activity.

AMP	No. of amino acids	Susceptible cancer cells	References
Pardaxin	33	Murine fibrosarcoma	[91]
Dermaseptin B2	33	Prostate adenocarcinoma cell line PC3	[92]
Magainins	21 to 27	HL-60 human promyelocytic leukemia cells, human lung carcinoma cells	[93]
Gaegurins	24	HCT116 colon and MCF-7 breast carcinoma cells	[86]
Melittin	26	U937 human monocytic leukemia cells, Du145 prostate carcinoma cells, SKOV3 ovarian carcinoma cells, B16 murine melanoma cells, BEL-7402 human hepatocellular carcinoma cells	[94, 95]
LL-37		Human oral squamous carcinoma cells, KB human squamous cancer cell lines	[96]
Cecropins	24–39	HL-60 human promyelocytic leukemia cells, CCRF-SB human lymphoblastic leukemia cells, EJ human bladder carcinoma cells, ascitic colon adenocarcinoma cells, bladder tumour cells lines, RT4, 647V, J82, 486P and benign fibroblast cell line, 3T6	[97–101]
BMAP-27, BMAP-28	27, 28	Human leukemia cells, CEM-CCRF human T leukemia cells, U937 and K562 human leukemia cell lines	[102]
Defensins	29 to 45	Raji human B-lymphoma cells, human oral squamous carcinoma cells, MOT mouse teratocarcinoma cells, fibroblast and epithelial cells	[103, 104]
Lactoferricin (Lfcin B)	25	Human leukemia and breast carcinoma cells, human endothelial cells	[105, 106]
Tachyplesin I	17	Human TSU prostate carcinoma cells, endothelial cells, B16 melanoma cells, SMMC-7721 human hepatoma cells, BGC-83 human gastric adenocarcinoma cells.	[107]
PR-39	39	Human hepatocellular carcinoma cell lines	[108]
Cecropin-Melittin (CA-ME)	20	Human small cell lung cancer cell line	[109]
Cecropin-Magainin (CA-MA)	20	Jurkat T leukemia cells, K562 chronic myeloid leukemia cells	[55]

**Table 5:** List of antimicrobial peptides as signalling molecules.

AMP	Source	Location	Function as signalling molecule	References
Human neutrophil peptides (HNP-1, HNP-2, and HNP-3)	Human	Bone marrow cells, peripheral leukocytes	Inhibitor of phospholipid/Ca^2+^ protein kinase (PKC), phosphorylation of endogenous proteins, chemo attractant of monocytes, stimulate release of cytokines (IL-1 and IL-8) and TNF	[121]
Beta defensins	Human, rabbit, guinea pig	Epithelial cells lining various organs such as epidermis, bronchial tree, and genitourinary tract	Induce release of histamine and prostaglandin 2 by activation and degranulation of mast cells	[122]
CAP37	Human	Polymorphonuclear leukocytes (PMNs), platelets, ocular epithelia	Chemotactic attractant for monocytes, binds heparin and LPS, induce leukocyte adhesion to endothelial cells, upregulate adhesion molecules, involved in leukocyte epithelial and epithelial extracellular matrix interactions, Upregulation of phospholipid/Ca^2+^ protein kinase (PKC)	[123]
PR-39	Pig	Intestine	Inhibit ubiquitin-proteasome-dependent degradation of hypoxia-inducible factor-1*α* protein and induce angiogenesis, binds with cas protein and regulates cell adhesion, migration and transformation, inhibit P13-kinase activity,	[124, 125]
Epidermoid carcinoma-derived antimicrobial peptide (ECAP)	Human	Tumour cells	Inhibit EGFR auto phosphorylation and leads to decreased activity of nonreceptor protein kinases belonging to different families such Syk, Lyn and PKCmu	[126]
LL-37	Human	Conjunctival and corneal epithelial cells	Chemotactic for monocytes, T-cells, neutrophils and mast cells, stimulate angiogenesis, stimulates IL-8 secretion, modulate dendritic cells differentiation, activator of extracytoplasmic function (ECF) sigma factors and regulates stress tolerance, keratinocytes apoptosis, anti-inflammatory and antiendotoxic effects	[123, 127]
Protegrin-1 (PG-1)	Porcine	Leukocytes	Activator of extracytoplasmic function (ECF) sigma factors and regulates stress tolerance	[128]
